# Customizable Scintillator of Cs_3_Cu_2_I_5_:2% In^+^@Paper for Large‐Area X‐Ray Imaging

**DOI:** 10.1002/advs.202304957

**Published:** 2023-10-23

**Authors:** Weiqing Chen, Ting Wang, Tianchi Wang, Jing Yu, Shuyi Yao, Wei Feng, Qingyuan Wang, Ling Huang, Xuhui Xu, Xue Yu

**Affiliations:** ^1^ School of Mechanical Engineering Institute for Advanced Materials Chengdu University Chengdu 610106 P. R. China; ^2^ Faculty of Materials Science and Engineering Key Laboratory of Advanced Materials of Yunnan Province Kunming University of Science and Technology Kunming Yunnan 650093 P. R. China; ^3^ College of Materials and Chemistry & Chemical Engineering Chengdu University of Technology Chengdu Sichuan 610059 P. R. China; ^4^ State Key Laboratory of Chemistry and Utilization of Carbon Based Energy Resources College of Chemistry Xinjiang University Urumqi 830046 P. R. China

**Keywords:** customizable scintillator, large‐area, optical crosstalk, perovskite@paper, physical scattering

## Abstract

High‐resolution X‐ray imaging is increasingly required for medical diagnosis and large‐area detection. However, the issues of scattering and optical crosstalk are limiting the spatial resolution of the indirect X‐ray imaging. In this study, a feasible and efficient strategy is proposed to in situ synthesize flexible Cs_3_Cu_2_I_5_:2%In^+^@paper as a superior scintillator film, which can be scaled up to an ultra‐large area of 4800 cm^2^. The as‐obtained Cs_3_Cu_2_I_5_:2%In^+^@paper performs a fascinating photoluminescence quantum efficiency up to 88.14%, a steady state light yield of 70169 photons/MeV, and spatial resolution of 15 lp mm^−1^. Moreover, the suppressed physical scattering and optical crosstalk of the corresponding film are demonstrated. Accordingly, this work explores a feasible fabrication of customizable scintillation films with large area for high‐resolution X‐ray detection.

## Graphical abstract

High‐resolution X‐ray imaging is increasingly required for medical diagnosis and large‐area detection. Following the “ancient cloth dyeing process”, a large‐area perovskite@paper scintillator is obtained via a one‐step in situ preparation. Accordingly, a multi‐dimensional customized X‐ray imaging with a high X‐ray detection limit and excellent spatial resolution is achieved. Herein, an industrial roll‐to‐roll production of the corresponding scintillator can be expected.

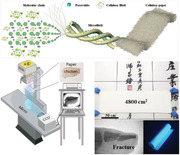

## 1 Introduction

Indirect X‐ray imaging with scintillator as the core component is widely utilized in many fields, such as medical imaging, security inspection, industrial flaw detection, and so on.^[^ ^1^, ^2^, ^3^, ^4^ ^]^ Especially in the view of the global coronavirus disease pandemic, medical X‐ray imaging with high‐resolution plays a significant role for detecting abnormal lung symptoms.^[^ ^5^, ^6^ ^]^ Accordingly, developing more convenient and cost‐saving X‐ray detector to obtain accurate information is an urgent need.^[^ ^4^ ^]^ Current researches focused on developing new scintillator materials,^[^ ^7^, ^8^ ^]^ simplifying the corresponding manufacturing procedure,^[^ ^9^ ^]^ designing the device structure,^[^ ^10^ ^]^ and thereby improving the resolution of X‐ray imaging^[^ ^11^, ^12^ ^]^ are underway. Although many successes have been achieved, the issues of scattering and optical crosstalk in X‐ray imaging have not been properly solved, which seriously affects the realization of high‐resolution imaging. It is mainly attributed to the following reasons. First, the refractive index of X‐ray is close to 1.0 for all materials and thereby it cannot be focused by lens.^[^ ^13^ ^]^ Hence, the scattering problem of the traditional projection X‐ray imaging is depicted in Figure S1 (Supporting Information). The physical distance between traditional flat‐panel detector and non‐planar objects causes uneven dose distribution of X‐rays spatially and edge scattering, resulting in the distorted image of the corresponding sample.^[^ ^2^ ^]^ It generally requires multiple consecutive exposures to obtain images with a sharp outline.^[^ ^14^, ^15^ ^]^ Second, the generated random secondary photons between the scintillator and the target object, interfering with the effective optical signals, resulting in optical crosstalk and a sharp drop in image contrast as well (Figure S1I, Supporting Information).^[^ ^16^, ^17^, ^18^ ^]^ Moreover, the irregularity of the target object and the expansion of the imaging field of view even aggravate the degree of image distortion significantly.^[^ ^19^, ^20^ ^]^ Basically, the complete attachment of the scintillation to the imaged object provides an effective way to solve light scattering and light crosstalk.^[^ ^7^ ^]^ As shown in Figure S1II (Supporting Information), a flexible scintillator film is supposed to minimize the effects of scattering and optical crosstalk via the attachment to the target. Hence, scintillator films with good X‐ray imaging performance as high light yield, high spatial resolution, along with a high flexibility less geared to the shape of an object are highly desired.

The low formation energy and superior X‐ray response of halide perovskite made exploring flexible perovskite scintillator films a fascinating study.^[^ ^21^, ^22^, ^23^ ^]^ The pre‐synthesized perovskite microcrystals could be directly embedded within polymethyl methacrylate, polydimethylsiloxane (PDMS), or other polymers to realize flexible applications.^[^ ^24^, ^25^, ^26^, ^27^, ^28^ ^]^ However, during the curing process of these polymers, there is an inevitable agglomeration of these crystals, which generally leads to a degraded performance of the as‐obtained films.^[^ ^23^ ^]^ Although in situ crystallization procedure is developed to form flexible perovskite composite films,^[^ ^20^, ^29^, ^30^ ^]^ the Ostwald ripening and agglomeration of the perovskite nanocrystals still cannot be overlooked, which not only increases the light scattering of the corresponding scintillator film, but also limits the loading concentration of the perovskite nanocrystal scintillators, leading to low scintillation output.

As a renewable materials, cellulose paper has excellent characteristics such as light weight, biocompatibility, feasible processing, and large‐area preparation, which is expected to be used as a flexible substrate.^[^ ^31^, ^32^, ^33^, ^34^ ^]^ Hence, we employ ordinary paper in this work, and follow the “ancient cloth dyeing process” to endow it scintillator characteristics via a one‐step preparation, thus realizing large‐area scintillator screen. Cs_3_Cu_2_I_5_:2%In^+^ crystals could be uniformly precipitated in situ on the cellulose paper after a feasible processing, and the processed cellulose paper could be folded and cut into objects of arbitrary shapes. Hence, it provides a multi‐dimensional customizable X‐ray imaging with a cost‐saving approach, and the scattering and optical crosstalk for the indirect X‐ray imaging could be greatly alleviated accordingly.

## 2 Results and Discussion

**Figure 1:**
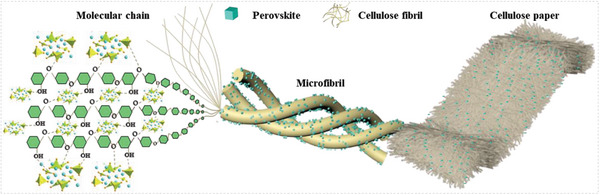
Schematic illustration of the cellulose fibrils bonding with the precipitated Cs_3_Cu_2_I_5_:2%In^+^ perovskite crystals.

Cellulose paper is composed of numerous cellulose nanofibrils with micro‐size, as the shown in the SEM (scanning electron microscope) images presented in Figure S2a–c (Supporting Information). It would provide abundant nucleation sites for the growth of the Cs_3_Cu_2_I_5_ perovskite. Moreover, due to the presence of abundant oxygen‐containing polar functional groups (eg, hydroxyl, ether bonds, etc.) in the cellulose molecular chain, it is suggested that the Lewis acid‐based coordination with Cu atoms promotes the uniform crystallization of Cs_3_Cu_2_I_5_:2%In^+^ (**Figure** **1**).

In this regard, a feasible two‐step preparation process, inspired by the ancient cloth dyeing method, is proposed for fabricating the Cs_3_Cu_2_I_5_:2%In^+^@paper (Figure S2d, Supporting Information). First of all, the precursor solution of Cs_3_Cu_2_I_5_:2%In^+^, CsI, CuI, and InI are dissolved separately in the *N*,*N*‐Dimethylformamide (DMF) solution. Then, the cellulose paper is immersed into the precursor solution. Simultaneously, the precursor ionic gradually diffused into the cellulose molecular chain based on the capillary effects. Subsequently, the soaked cellulose paper was transferred into a baking oven and heat‐treated at 323 K for 30 mins. Finally, the Cs_3_Cu_2_I_5_:2%In^+^@paper with an area up to 4800 cm^2^ is believed to have been successfully prepared.

**Figure 2:**
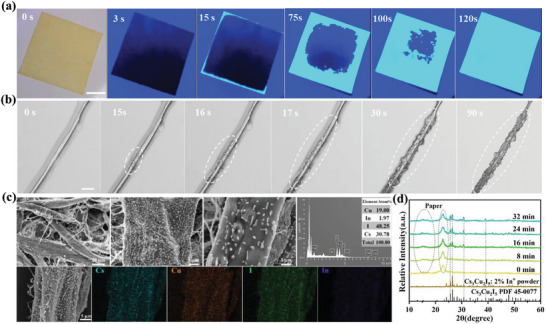
a) Photographs of the cellulose paper with the embedded Cs_3_Cu_2_I_5_:2%In^+^ recorded from 0 to 120 s under 254 nm UV illumination at a plate heater of 323 K. Scale bar is 5 mm. b) In situ microscopic image of the crystallization process of Cs_3_Cu_2_I_5_:2%In^+^ growth at a single cellulose fiber surface. Scale bar is 50 µm. c) SEM image, energy dispersive spectroscopy, and the corresponding elemental mapping of the as‐obtained Cs_3_Cu_2_I_5_:2%In^+^@paper sample. d) X‐ray diffraction patterns of Cs_3_Cu_2_I_5_:2%In^+^@paper recorded with a prolonged time.

To investigate the in situ formation of Cs_3_Cu_2_I_5_:2%In^+^@paper, photographs of the cellulose paper with the in situ formed Cs_3_Cu_2_I_5_:2%In^+^ were recorded as shown in **Figure** **2**a. As the DMF solvent gradually evaporates, a bright blue light emerges from the edges of the cellulose paper and gradually spreads, eventually covering the entire cellulose paper evenly. It is speculated that this phenomenon is attributed to the diffusion of the precursor ions and the effective coordination between Cu^+^ and cellulose molecular chains (Figure S3, Supporting Information). Additionally, the in situ microscopy images of the crystallization process of Cs_3_Cu_2_I_5_:2%In^+^ was recorded using an optical microscopy (Figure 2b). Initially, a lay of the precursor solution coating on the surface of cellulose fiber is observed, and crystals gradually precipitate from the surface of the fiber, and growth along with the evaporation of the solvent, while eventually covering the whole fiber. The corresponding luminescence photograph given in Figure S4 (Supporting Information) displays the bright bule emission, which is consistent with the characteristic emission of Cs_3_Cu_2_I_5_:2%In^+^.

The SEM image of the formed Cs_3_Cu_2_I_5_:2%In^+^@paper are given in Figure 2c, which reveals that the Cs_3_Cu_2_I_5_:2%In^+^ microcrystalline particles with excellent crystallinity are uniformly distributed on the fiber surface. The corresponding energy dispersive spectroscopy mapping image manifests the homogeneous distribution of Cs, Cu, I, and In elements in the precipitated particles. Moreover, in situ X‐ray diffraction patterns of Cs_3_Cu_2_I_5_:2%In^+^@paper were carried out for confirming the formed phase (Figure 2d). At 0 min, only two wide diffraction peaks of cellulose paper appeared. With the extension of the recorded time, several characteristic diffraction peaks gradually emerge and match well with the diffraction peaks of Cs_3_Cu_2_I_5_:2%In^+^, confirming the successful precipitation of the Cs_3_Cu_2_I_5_ microcrystalline particles from the cellulose paper surface. It is worth noting that the average diameter of the formed Cs_3_Cu_2_I_5_:2%In^+^ grains is ≈1–3 µm. Interestingly, this in situ preparation method is universal, and can also be utilized to prepare the Cs_3_Cu_2_Cl_5_@paper, Cs_3_Cu_2_Br_5_@paper (Figure S5, Supporting Information).

**Figure 3:**
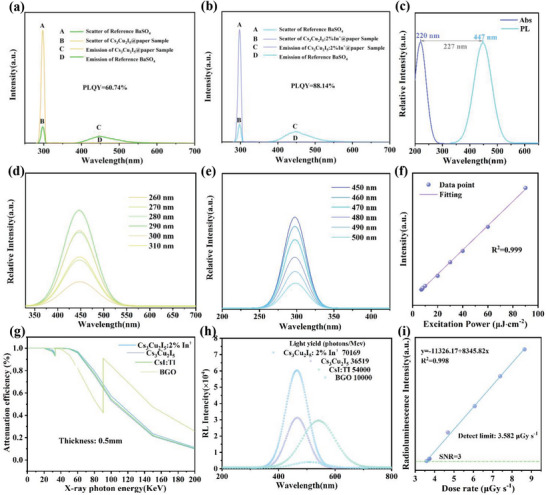
Photoluminescence quantum yield evaluation of a) Cs_3_Cu_2_I_5_@paper and b) Cs_3_Cu_2_I_5_:2%In^+^@paper collected by an integrated sphere, respectively. c) UV–vis absorption and photoluminescence spectra of the Cs_3_Cu_2_I_5_:2%In^+^@paper. d) PL spectra of the Cs_3_Cu_2_I_5_:2%In^+^@paper recorded under different excitation wavelengths. e) PLE spectra of the Cs_3_Cu_2_I_5_:2%In^+^@paper monitored at different emission wavelengths. f) The plotted PL intensity of Cs_3_Cu_2_I_5_:2%In^+^@paper as a function of pump density. g) X‐ray absorption spectra and h) RL spectra of the Cs_3_Cu_2_I_5_:2%In^+^@paper, Cs_3_Cu_2_I_5_@paper, CsI:Tl, and BGO, respectively. i) RL intensity of Cs_3_Cu_2_I_5_@paper as a function of dose rate.

A series of In^+^ doped Cs_3_Cu_2_I_5_ samples have been fabricated to further optimize the crystal structure of Cs_3_Cu_2_I_5_, and the corresponding phase patterns are given in Figure S6 (Supporting Information). The characteristic diffraction peaks of Cs_3_Cu_2_I_5_ become sharped gradually with the introduction of In^+^ ions, confirming the successful improvement of the crystallinity of the Cs_3_Cu_2_I_5_ crystals. Accordingly, the X‐ray photoelectron spectroscopy curves of the In^+^ doped Cs_3_Cu_2_I_5_, given in Figure S7 (Supporting Information), prove that In^+^ ions were successfully incorporated into Cs_3_Cu_2_I_5_ crystals. Moreover, the corresponding photoluminescence quantum yield (PLQY) of Cs_3_Cu_2_I_5_ is significantly improved from 60.74% to 88.14% thanks to the introduction of In^+^ ions (**Figure** **3**a,b). Additionally, the large stokes shift of 227 nm significantly reducing the self‐absorption effect is observed as depicted in Figure 3c. As exhibited in Figure 3d,e, the photoluminescence (PL) and photoluminescence excitation (PLE) spectra shows identical shape and peak position. Moreover, the PL intensity is linearly correlated with excitation power, indicating the exclusion of the permanent defects’ contribution (Figure 3f).^[^ ^35^ ^]^ It supports that the self‐trapped exciton is the emission mechanism of Cs_3_Cu_2_I_5_:2%In^+^@paper. The results based on the photon cross‐section database are shown in Figure 3g. Cs_3_Cu_2_I_5_:2%In^+^@paper also exhibits excellent X‐ray absorption properties (Figure 3g). Furthermore, the radioluminescence (RL) spectra of Cs_3_Cu_2_I_5_:2%In^+^@paper was estimated (Figure 3h). In order to ensure the relative accuracy of the measurement of light yield, we adopt the method of cross‐contrast test. Under the same experimental conditions, commercial BGO and CsI:Tl were employed as reference objects. By comparing the integral area of RL spectrum, the steady‐state light yield of Cs_3_Cu_2_I_5_:2%In^+^@paper is as high as 70169 photons/MeV, which is comparable to commercial CsI:Tl. It could be attributed to its high PLQY, negligible self‐absorption of the corresponding Cs_3_Cu_2_I_5_:2%In^+^@paper scintillator. In addition, the detection limit of Cs_3_Cu_2_I_5_:2%In^+^@paper also reached 3.582 uGy s^−1^ as shown in the Figure 3i, which meets the requirements of X‐ray medical diagnosis (5.5 µGy s^−1^).

To improve the chemistry stability of the as‐obtained Cs_3_Cu_2_I_5_:2%In^+^@paper, the Cs_3_Cu_2_I_5_:2%In^+^@paper was coated with PDMS, as given in Figure S8 (Supporting Information). Notably, the hydrophobic angle of the composite cellulose paper reaches 129°, and the corresponding treated sample exhibits a superior stability. After the sample deposited in a 45% humidity environment for 200 days, the light intensity of the packaged paper is hardly decreased, showing excellent stability. Additionally, the irradiated photostability of the Cs_3_Cu_2_I_5_:2%In^+^@paper is further examined under continuous and repeated X‐ray irradiation cycles. As shown in Figure S9 (Supporting Information), at a dose rate of 600 µGy s^−1^ for 100 min and 100 on‐off cycles, the change in RL intensity is negligible. In addition, after continuous irradiation for 820 min at a dose rate of 4.5 mGy s^−1^, the RL intensity remains above 98%, which confirmed its excellent anti‐irradiation ability.

**Figure 4:**
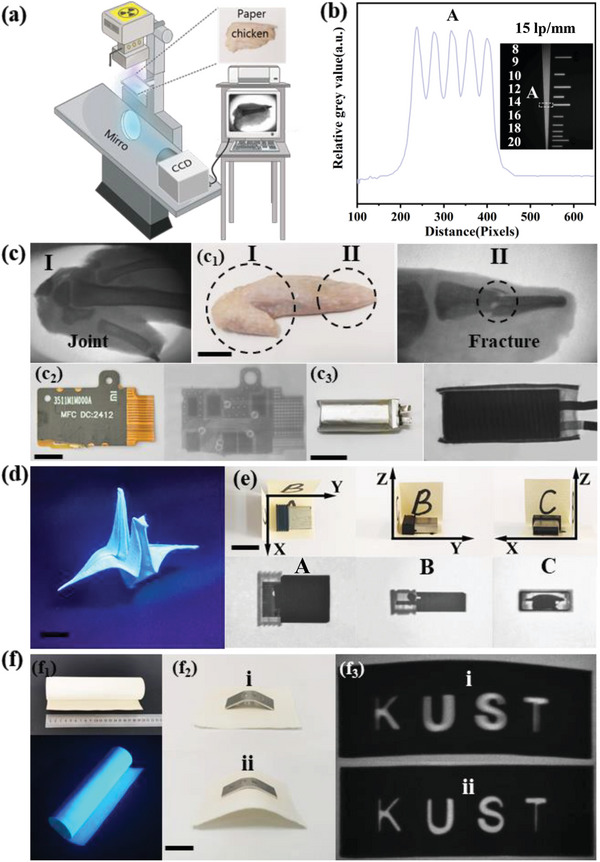
a) Schematic diagram of the as‐designed indirect X‐ray imaging system. b) Gray value distribution extracted from the dotted box of the resolution card (the inset (b)). The inset is the corresponding X‐ray image with the Cs_3_Cu_2_I_5_:2%In^+^@paper of the standard X‐ray resolution pattern plate (dose rate: 600 µGy s^−1^, voltage: 30 kV). Numbers 8–20 indicate the corresponding spatial resolution as lp mm^−1^. c) Demo X‐images of c_1_) a chicken wing (Scale bar is 15 mm), c_2_) a chip (Scale bar is 4 mm), and c_3_) a lithium battery (Scale bar is 1.6 mm), respectively (dose rate: 600 µGy s^−1^, voltage: 30 kV). d) Photographs of a folded crane of the as‐obtained paper under 254 nm UV light (Scale bar is 10 mm). e) X‐ray images of the target captured from top, front, and sided view (Scale bar is 10 mm), respectively (dose rate: 600 µGy s^−1^, voltage: 30 kV). f) Photographs of f_1_) the curl paper under sunlight and 254 nm UV light, respectively; f_2_) Photographs and the f_3_) corresponding X‐ray images of a curved iron plate with hollowed‐out characters of KUST in a i) projection and ii) attached X‐ray imaging approach (Scale bar is 5 mm), respectively (dose rate: 400 µGy s^−1^, voltage: 30 kV).

Moreover, Figure S10 (Supporting Information) illustrates that the optical transparency of Cs_3_Cu_2_I_5_:2%In^+^@paper is improved, which is beneficial for X‐ray imaging. The imaging performance of the samples was characterized by using a self‐built imaging platform (**Figure** **4**a). As shown in Figure 4b, the value of the spatial resolution of the standard test‐pattern plate is determined to be 15 lp mm^−1^. Furthermore, the X‐ray images show the detailed fracture (II) information of chicken wing bone as depicted in Figure 4c. The wiring information inside the chip and lithium battery can be clearly captured under X‐ray irradiation as well. In addition, the as‐prepared Cs_3_Cu_2_I_5_:2%In^+^@paper exhibits a foldable characteristic (Figure 4d). Thus, a multidimensional imaging was designed to capture the internal structure information of the USB from three observation angles. Figure 4e clearly shows the internal structure information of the USB from multiple perspectives.

As shown in Figure 4f, the Cs_3_Cu_2_I_5_:2%In^+^@paper exhibits excellent flexibility and can fit perfectly with the imaged object. The flexible scintillators can solve the scattering problem of imaging curved objects superior to that of flat scintillators. As shown in Figure 4f, the KUST imaged by the projection imaging has ghosting phenomenon, while the flexible scintillator can perfectly fit the imaged object, and the corresponding imaged KUST has a clear outline without ghosting (as shown in the below Figure 4f_3_). In addition, the flexible imaging properties of the Cs_3_Cu_2_I_5_:2%In^+^@paper are further characterized in Figures S13 and S14 (Supporting Information).

**Figure 5:**
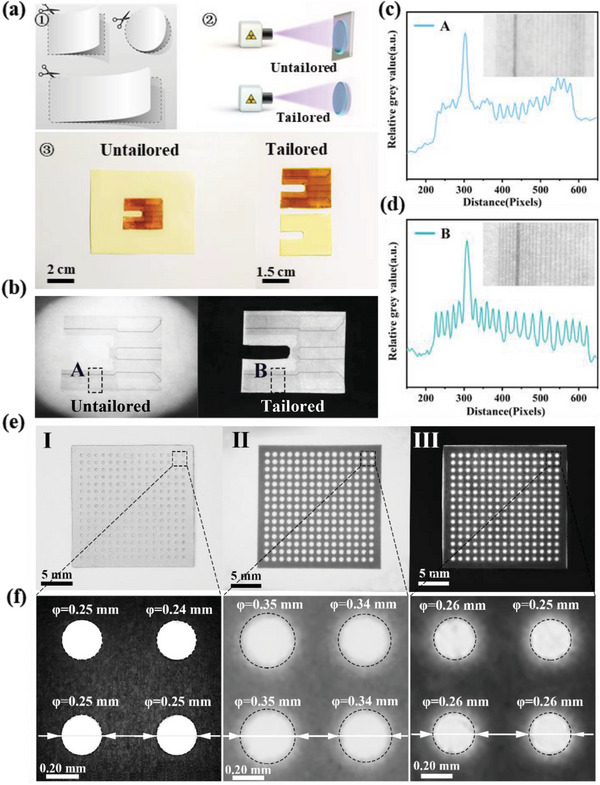
a) Schematic diagram of the untailored and tailored imaging for a U‐shaped circuit board and b) the corresponding X‐ray images (dose rate: 400 µGy s^−1^, voltage: 30 kV). The modulated converted image of the partial imaging of the region c) A and d) B in inset (b). e) Physical map of a mask plate (I), untailored (II), and tailored X‐ray image (III) of the corresponding mask (dose rate: 400 µGy s^−1^, voltage: 30 kV). f) The corresponding partial magnified image of inset (e).

The paper can be easily cut into arbitrary shape, which could match the corresponding target object perfectly. Accordingly, Cs_3_Cu_2_I_5_:2%In^+^@paper is utilized to achieve customized imaging, alleviating the effects of scattering and lateral optical crosstalk, which is similar to the process of reducing the imaging field of vision.^[^ ^16^ ^]^ As demonstrated in Figure S15 (Supporting Information), the scattering effect gradually increases as the irradiation area increases. A comparative experiment with different imaging approach as shown in **Figure** **5**a was designed. U‐shaped circuit board is used as an imaging object, while untailored and tailored imaging approaches are employed. The corresponding imaging photograph with a untailored Cs_3_Cu_2_I_5_:2%In^+^@paper is shown in Figure 5b (left), while the image with the Cs_3_Cu_2_I_5_:2%In^+^@paper scintillator trimmed into a ratio of 1:1 to the target is displayed in Figure 5b (right), respectively. The corresponding partial image captured at the region at A and B in Figure 5b is further depicted in Figure 5c,d, respectively, which confirms that the image B has a sharper contrast. The main reason is that, under X‐ray irradiation, the optical channel crosstalk may lead to a reduction in the transmitted efficiency of the RL and a decrease in X‐ray imaging resolution. In order to further quantify the phenomenon and effect of cropped imaging, the reticle is used as the imaging object. Compared with the aperture diameter of reticle I checked under the microscope in Figure 5e, the aperture diameter of reticle I is ≈0.25 mm. The aperture of the uncropped image II is severely distorted, reaching as large as 0.35 mm, while the aperture of the cropped image III is determined to be 0.26 mm. Hence, the excellent processability of the flexible Cs_3_Cu_2_I_5_:2%In^+^@paper as a scintillator indicates a fascinating X‐ray imaging could be achieved convenient and cost‐saving.

## 3 Conclusion

In conclusion, flexible Cs_3_Cu_2_I_5_:2%In^+^@paper scintillation screens with an area of up to 4800 cm^2^ were prepared by a simple soaking and drying process. The flexible and tailorable properties of the cellulose paper are harnessed as a superior scintillator, effectively solving the problems of scattering and optical crosstalk during indirect X‐ray imaging. The Cs_3_Cu_2_I_5_:2%In^+^@paper scintillator possesses a high steady‐state X‐ray light yield of 70169 photons/MeV and high spatial resolution of 15 lp mm^−1^, it can meet the requirements of flat, flexible, and customized X‐ray scintillators, matching multi‐scene X‐ray imaging. In addition, the characteristics of the fiber‐based paper can be well coupled to the future industrial roll‐to‐roll production. It is expected to advance the industrialization of low‐cost large‐area flexible perovskite scintillator screens.

## Conflict of Interest

The authors declare no conflict of interest.

## Supporting information

Supporting Information

Supplemental Video 1

Supplemental Video 2

## Data Availability

Research data are not shared.
