# Supplementary material for: Customizable Scintillator of Cs3Cu2I5:2% In+@Paper for Large‐Area X‐Ray Imaging
Source: Adv Sci (Weinh). 2023 Oct 23;10(34):2304957. doi: 10.1002/advs.202304957 (PMC10700220; doi:10.1002/advs.202304957)
Supplement: Supplementary file 1 — Supporting Information [file ADVS-10-2304957-s002.pdf]

ADVANCED SCIENCE
Open Access

Supporting Information

for *Adv. Sci.*, DOI 10.1002/advs.202304957

Customizable Scintillator of $Cs_3Cu_2I_5$:2% In^+@Paper for Large-Area X-Ray Imaging

Weiqing Chen, Ting Wang, Tianchi Wang, Jing Yu, Shuyi Yao, Wei Feng, Qingyuan Wang, Ling Huang, Xuhui Xu and Xue Yu**

Supporting Information

Weiqing Chen[a, c], Ting Wang[b], Tianchi Wang[c], Jing Yu[c], Shuyi Yao[c], Wei Feng[a], Qingyuan Wang[a], Ling Huang[d], Xuhui Xu[c], Xue Yu[a*]*

[a] *School of Mechanical Engineering, Institute for Advanced Materials, Chengdu University, Chengdu, 610106, P. R. China.*

[b] *College of Materials and Chemistry & Chemical Engineering, Chengdu University of Technology, Chengdu, 610059, Sichuan, P. R. China.*

[c] *Faculty of Materials Science and Engineering, Key Laboratory of Advanced Materials of Yunnan Province, Kunming University of Science and Technology, Kunming, 650093, Yunnan, P. R. China.*

[d] *State Key Laboratory of Chemistry and Utilization of Carbon Based Energy Resources, College of Chemistry, Xinjiang University, Urumqi, 830046, P. R. China.*

* Corresponding Author: Xue Yu, E-mails: yuyu6593@126.com

Xuhui Xu, E-mails: xuxuh07@126.com

Experimental Section

Materials

Cesium iodide (CsI, 99.9%, Aladdin), caesium bromide (CsBr, 99.9%, Aladdin), cesium chloride (CsCl, 99.9%, Aladdin), copper iodide (CuI, 99.95%, Aladdin), cuprous bromide (CuBr, 99.99%, Aladdin), cuprous chloride (CuCl, 99.99%, Aladdin), indium(I) iodide (InI, 99.99%, Aladdin), N,N-dimethylformamide (DMF, 99.80%,

Aladdin), Cellulose paper (Dongguan Mingfang Paper Co., Ltd.). All raw materials were used as received without further purification.

Preparation of $Cs_3Cu_2I_5$:xIn$^+$@paper, (x=0, 1%, 2%, 3%, 4% and 5%)

To prepare undoped $Cs_3Cu_2I_5$@paper film: 15 mmol CsI, 10 mmol CuI and 20 mL DMF were strring at 80 °C for 30 minutes. The precursor solution was transferred to a petri dish, and the cellulose paper was soaked in the precursor solution for 1 h. Then the soaked cellulose paper is dried on a baking oven. After the solvent volatilization was completed, the $Cs_3Cu_2I_5$@paper with a certain thickness could be obtained. For the $Cs_3Cu_2I_5$:xIn$^+$ @paper film (x= 1%, 2%, 3%, 4% and 5%): InI were employed into the above precursor solution as CuI: InI=9.9: 0.1, 9.8: 0.2, 9.7: 0.3, 9.6: 0.4, and 9.5: 0.5, respectively.

Preparation of $Cs_3Cu_2Br_5$@paper and $Cs_3Cu_2Cl_5$@paper

To prepare $Cs_3Cu_2Br_5$@paper film: 15 mmol CsBr, 10 mmol CuBr and 20 mL DMF were strring at 80 °C for 30 minutes. The precursor solution was transferred to a petri dish, and the cellulose paper was soaked in the precursor solution for 1 h. Then, the soaked cellulose paper is dried on a baking oven. After the solvent volatilization was complete, the $Cs_3Cu_2Br_5$@paper with a certain thickness could be obtained. To prepare $Cs_3Cu_2Cl_5$@paper film: above 15 mmol CsBr and 10 mmol CuBr were replaced by 15 mmol CsCl, 10 mmol CuCl, respectively.

Material Characterization

Fourier transform infrared (FTIR) spectra was carried out using a Bruker ALPHA FTIR spectrometer. X-ray diffraction (XRD) patterns were recorded on a Bruker /D8

FOCUS X-ray diffractometer with Cu-Kα radiation source (wavelength at 1.5405 Å). The photoluminescence (PL) spectra of the samples were recorded on Hitachi fluorescence spectrometer F-7000. Transient PL decay properties measurements were performed by an Edinburgh FLS980 spectrometer. PLQY was estimated using an Edinburgh FLS 980 spectrometer equipped with an integrating sphere. SEM images and elemental mapping analysis were captured with a JEOL Field Emission SEM. X-ray Photoelectron Spectroscopy (XPS) were recorded by a Thermo Fisher Scientific K-Alpha and X-ray source (monochromatic Al Kα source (Mono Al Kα) energy: 1486.6 eV, 6 mA × 12 KV) with a 400 μm spot size and 50 eV pass energy.

In-Situ optical microscopy imaging

First of all, the cellulose soaked into the precursor solution was placed on a microscope slide. The samples were placed on the X-Y-Z translation stage of a bright-field optical microscope (Leica DM1000 LED) for optical (PL and laser spectroscopy) characterization. 40×objective lens were used to observe the topography of the sample. The in-situ crystallization process of microcrystalline particles on the cellulose surface was recorded in an ambient environment with the volatilization of the solvent.

X-ray imaging characterization

The X-ray detection equipment in this work is a D8 focused diffractometer (Bruker) with Cu-Kα radiation (λ= 0.15405 nm) and a commercially available small X-ray tube (Amptek) with a maximum output of 1200 W. The X-ray dose rate of the X-ray source can be controlled from 50 nGy s^{-1} to 8 mGy s^{-1} by adjusting the current and

voltage. For the measurement of stability under X-ray irradiation, the X-ray tube voltage was maintained to be 40 kV and 30 mA. The X-ray-induced luminescence photos were taken with a digital camera (Nikon D7100 with AF-S Micro-Nikkor 105 mm f / 2.8G IF-ED). In addition, in order to eliminate the negative impact of the direct radiation of the X-ray source on the camera, a mirror is used to deflect the optical path by 90°.The emission spectra under the X-ray irradiation of the samples were recorded by a FLAME-S-XR1-ES spectrophotometer (Ocean Optics USB2000+XR1-ES, corporate agent: Shenzhen Yaneo Instrument Ltd, Shenzhen, China).

Estimation of the light-yield

To estimate the light yield of $Cs_3Cu_2I_5$:2% In^+@paper and $Cs_3Cu_2I_5$@paper, $Bi_4Ge_3O_{12}$ (BGO) and CsI: Tl powders were employed as the reference scintillators. To unify the absorbed X-ray energy of these scintillator, the BGO and CsI: Tl powders were pressed into wafers with the same thickness and sectional area, as well as the same shape. These scintillators were then closely attached to the circular window of an integrating sphere, with a fixed distance to the X-ray source. The corresponding RL spectra were recorded by using a USB2000+XR1-ES fiber-coupled fluorescence spectrometer, which was carefully calibrated by an Ocean Optics engineer before usage. By comparing the integrated intensities of the four spectra, the light yield of 36519 and 70169 photons/MeV for the $Cs_3Cu_2I_5$@paper and $Cs_3Cu_2I_5$:2% In^+@paper scintillator was acquired, respectively.

The X-ray attenuation efficiency (AE) could be calculated using the following formula:

$$AE(\%) = (1 - e^{-c(E)\rho d}) \times 100\%$$

where c(E) is the photon cross section function obtained from the XCOM database of National Institute of Standards and Technology (NIST), E is the corresponding photon energy, ρ is the density of scintillator and d is the thickness of the scintillator film.

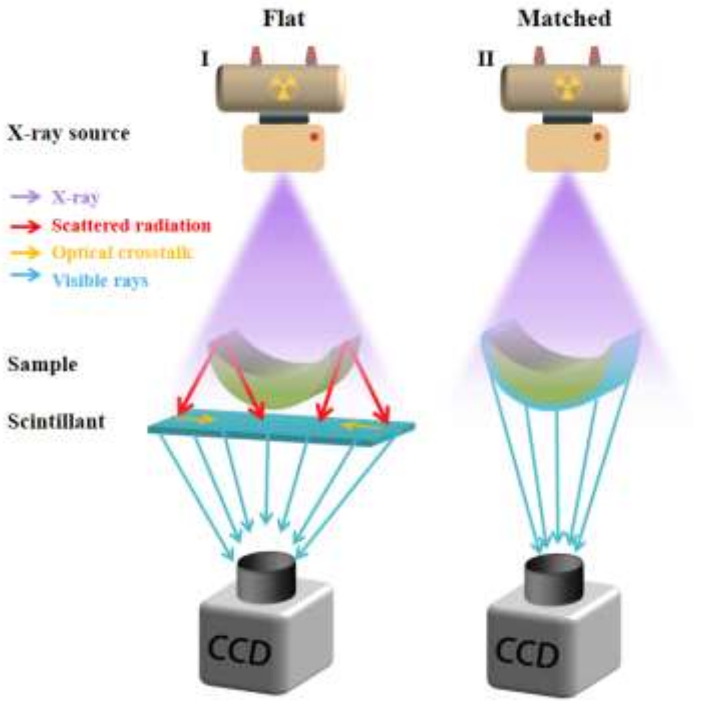

Figure S1. Schematic diagram of indirect X-ray imaging with distinct modes. I) Flat-panel projected, and II) perfect attached imaging, respectively.

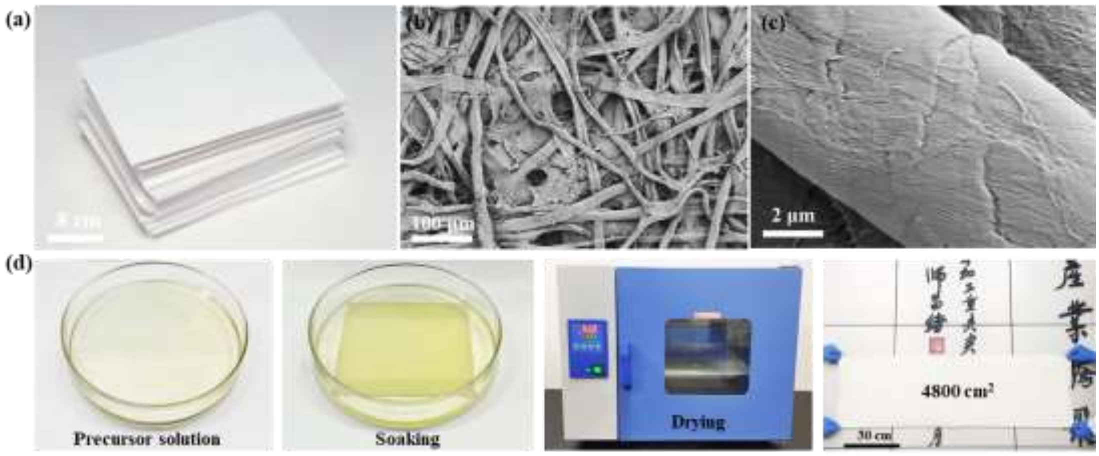

Figure S2. (a) Photograph and (b) the corresponding SEM image of the cellulose

paper, and (c) the SEM image of a cellulose micro fibril. (d) The *in-situ* fabrication process of $Cs_3Cu_2I_5$:2%In^+@paper.

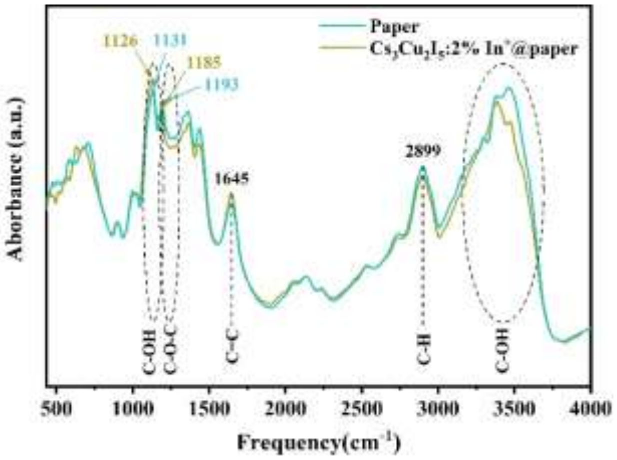

Figure S3. Fourier infrared absorption spectra of $Cs_3Cu_2I_5$:2%In^+@paper and pure cellulose paper, respectively. Compared with the pure paper, the vibrational peaks of C-OH and C-O-C of $Cs_3Cu_2I_5$:2%In^+@paper shift from 1131 cm^{-1} to 1126 cm^{-1} and from 1193 cm^{-1} to 1185 cm^{-1}, respectively. It is due to the interaction between the Lewis acid Cu atom and the Lewis base O atom.

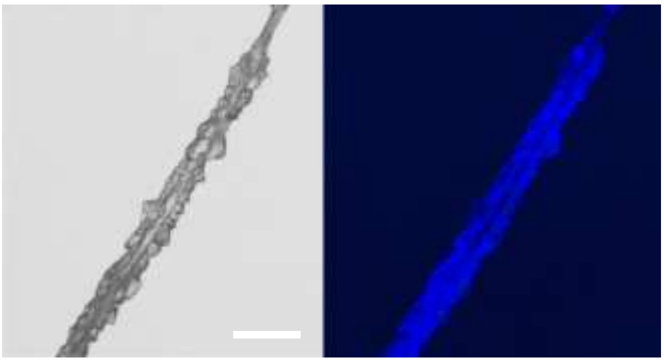

Figure S4. Photographs of a single fiber recorded with an optical microscope. Scale bar is 80 μm.

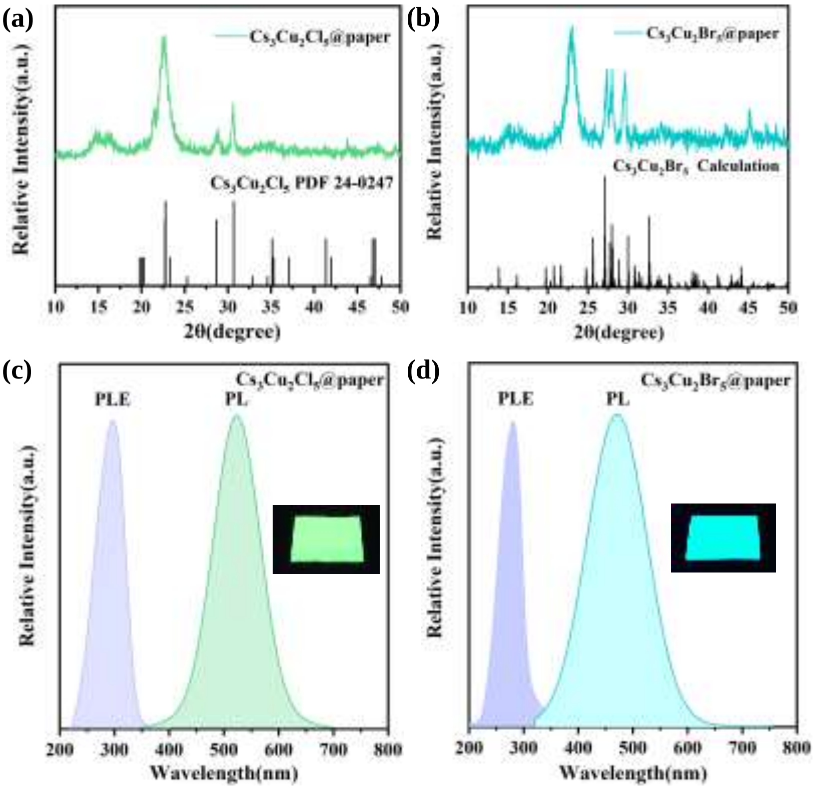

Figure S5. (a) XRD patterns of $Cs_3Cu_2Cl_5$@paper and (b) $Cs_3Cu_2Br_5$@paper, respectively. (c) UV-Visible excitation spectrum and photoluminescence spectra of the $Cs_3Cu_2Cl_5$@paper, and (d) $Cs_3Cu_2Br_5$@paper, respectively.

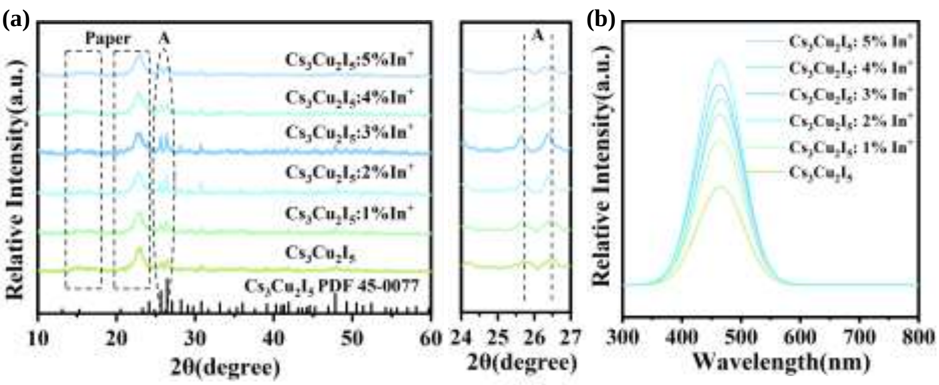

Figure S6. (a) XRD patterns and (b) Radioluminescence spectrum of $Cs_3Cu_2I_5:xIn^+$ (x= 0, 0.01, 0.02, 0.03, 0.04, and 0.05), respectively.

As shown by A in Fig. S3a, the characteristic diffraction peaks of 25.6° and 26.3°

blue-shifts with the increase of doping concentration. It is attributed to the larger ionic radius of In^+ than that of Cu^+ ions, resulting in the lattice expansion. The intensity of RL was enhanced due to the addition of heavy atoms. When the doping concentration of In^+ ions is 2%, the luminescence intensity of RL reaches the maximum.

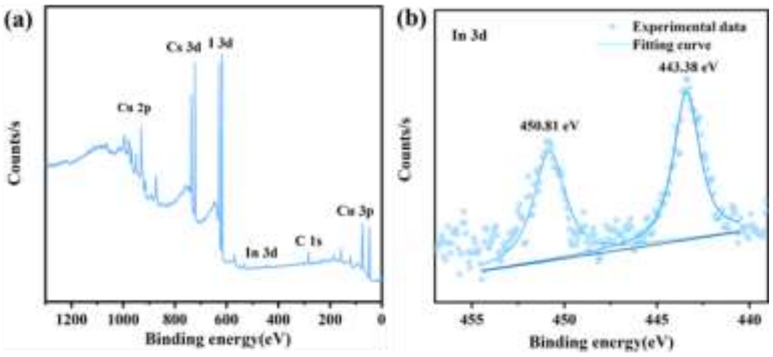

Figure S7. (a) XPS survey spectrum of $Cs_3Cu_2I_5$:2%In^+. (b) The XPS spectrum of the 3d orbit of In^+ with the binding energy of 443.38 eV and 450.81 eV, proving the successful introduction of In^+ ions.

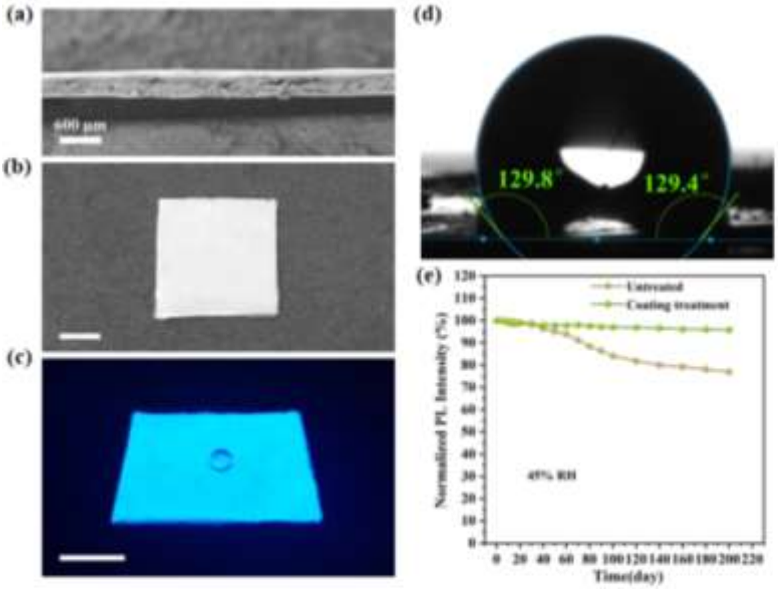

Figure S8. (a) SEM image of the coated $Cs_3Cu_2I_5$:2%In^+@paper. (b) Photograph of

the coated $Cs_3Cu_2I_5$:2%In^+@paper. Scale bar is 10 mm. (c) Photograph of the water droplets on the surface of paper under 254nm UV light. Scale bar is 10 mm. (d) The apparent contact angle of water droplet of $Cs_3Cu_2I_5$:2%In^+@paper. (e) Long-term stability of $Cs_3Cu_2I_5$:2%In^+@paper before and after the coating treatment at 45% humidity.

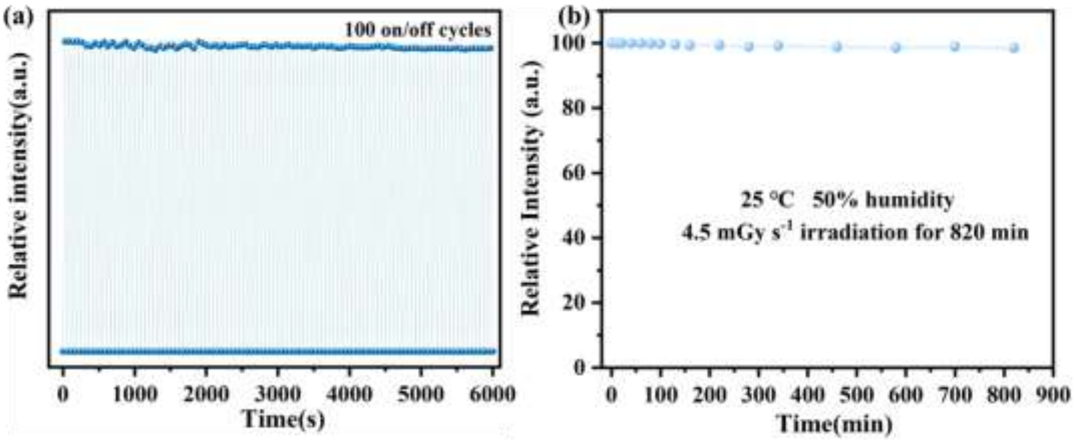

Figure S9. (a) RL intensity recorded with and without X-ray irradiation of the $Cs_3Cu_2I_5$:2%In^+@paper for 100 cycles continuously (dose rate: 600 μGy s^{-1}, voltage: 30 kV). (b) RL intensity recorded under continuous X-ray irradiation of the $Cs_3Cu_2I_5$: 2%In^+@paper.

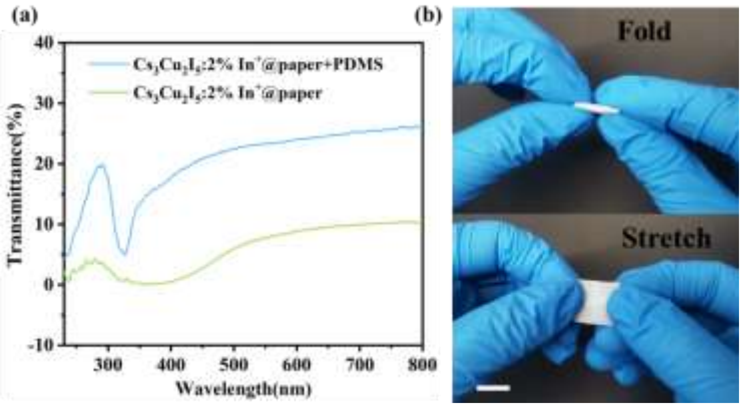

Figure S10. (a) Transmittance spectra of $Cs_3Cu_2I_5$:2%In^+@paper before and after

coating treatment. (b) Photographs recorded for bending and stretching of $Cs_3Cu_2I_5$:2%In^+@paper. Scale bar is 20 mm.

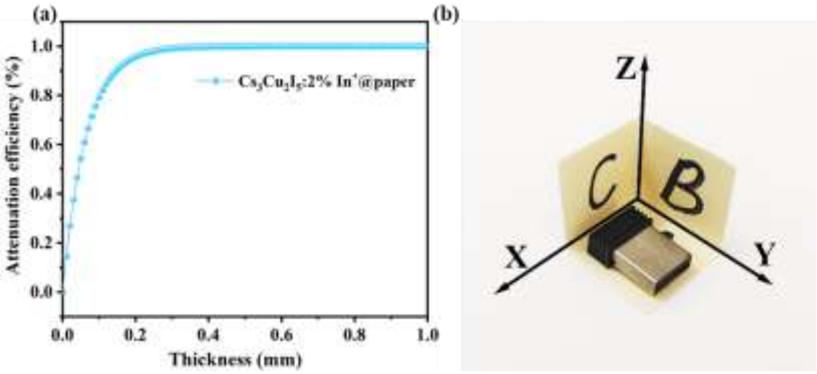

Figure S11. (a) X-ray absorption spectra measured as a function of X-ray energy of cellulose paper with different thickness. (b) Multi-dimensional imaging for a target.

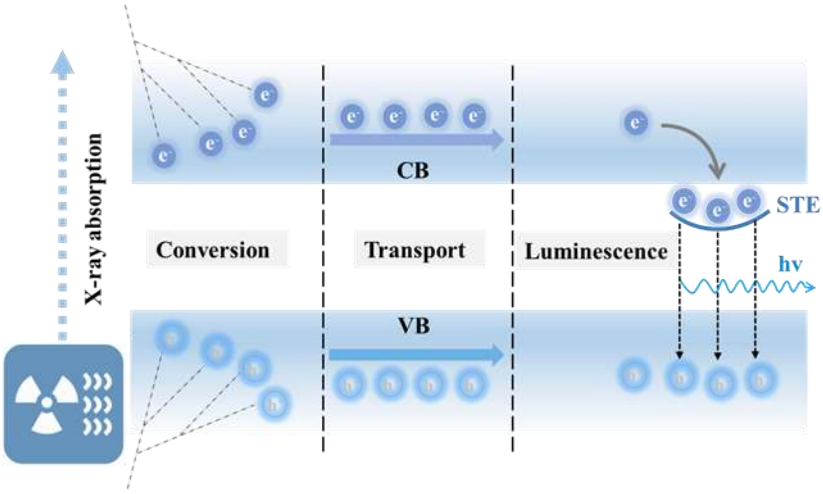

Figure S12. Radiation luminescence mechanism diagram of $Cs_3Cu_2I_5$:2%In^+@paper.

The detection mechanism of $Cs_3Cu_2I_5$:2%In^+@paper is based on a band model, as depicted in the figureS12. Upon high-energy X-ray radiation, $Cs_3Cu_2I_5$:2%In^+ is ionized to produce electron-hole pairs. This process leads to the accumulation of holes in the valence band and electrons in the conduction band. Subsequently, these

electrons and holes are transported to the light-emitting center. Finally, the electrons combine with holes through the STE (Self-trapping emission mechanism) process,[1-5] resulting in the emission of blue emission.

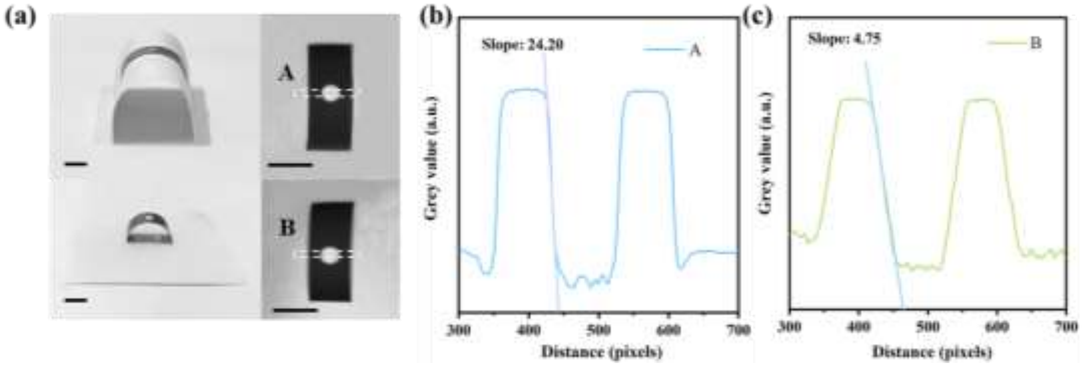

Figure S13. (a) Photographs and the corresponding X-ray images of $Cs_3Cu_2I_5$:2%In^+@paper in an attached and projected way. Scale bar is 5 mm (dose rate: 400 μGy s^{-1}, voltage: 30 kV). (b) (c) The correspondingly contour profiles of the partial image in (a) image.

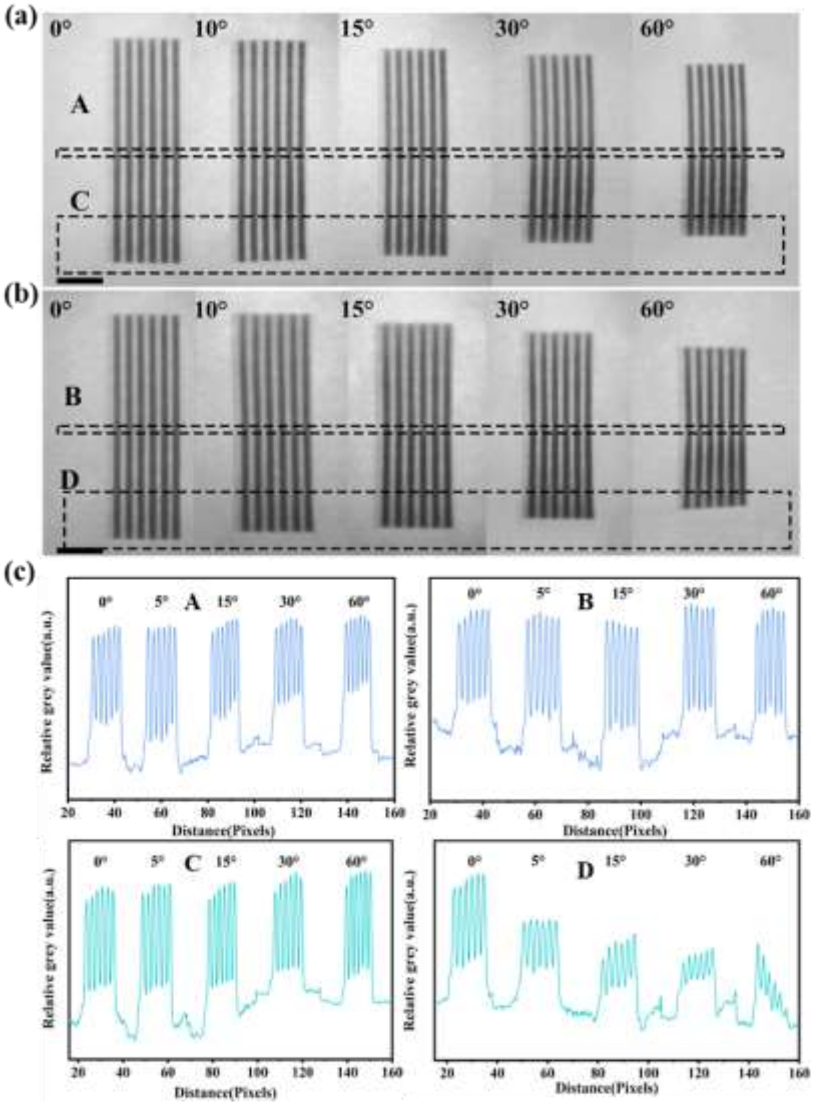

Figure S14. The indirect X-ray imaging with a flexible scintillator via (a) an attached and (b) a contactless way, respectively, the corresponding images were recorded for imaged object bended with different angle, Scale bar is 5 mm (dose rate: 400 μGy s^{-1}, voltage: 30 kV). (c) The corresponding contour profiles of bar pattern phantom.

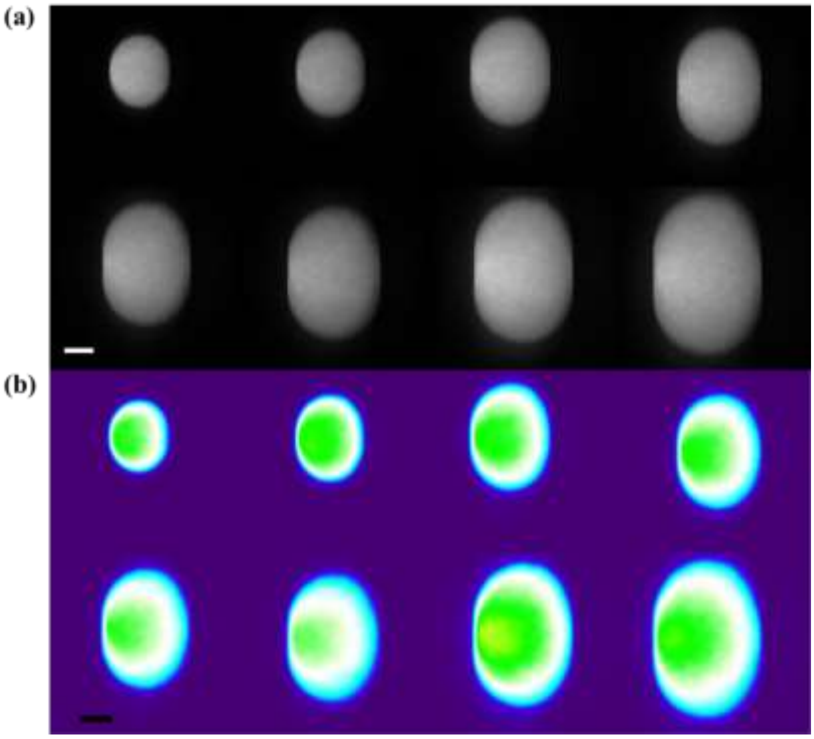

Figure S15. (a) Irradiance luminescence images with the increased irradiated area, and (b) the corresponding pseudo-color images. Scale bar is 10 mm.

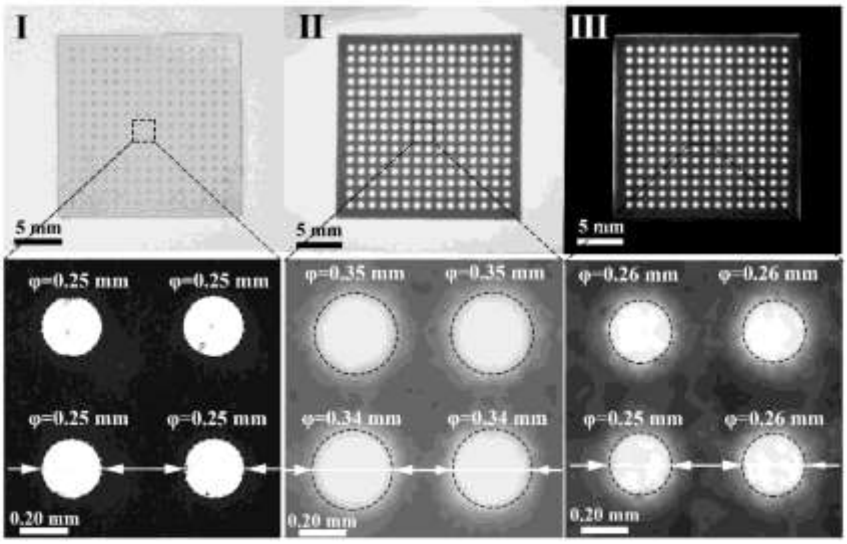

Figure S16. **I** Physical map of a mask plate, **II** untailored, and **III** tailored X-ray image of the corresponding mask (dose rate: 400 μGy s^{-1}, voltage: 30 kV), and the corresponding partial magnified image.

Reference

[1] O. D. I. Moseley, T. A. S. Doherty, R. Parmee, M. Anaya, S. D. Stranks, *J. Mater. Chem. C.* **2021,** 9, 11588.

[2] Y. Zhou, J. Chen, O. M. Bakr, O. F. Mohammed, *ACS Energy Lett.* **2021,** 6, 739.

[3] L. Lian, M. Zheng, W. Zhang, L. Yin, X. Du, P. Zhang, X. Zhang, J. Gao, D. Zhang, L. Gao, G. Niu, H. Song, R. Chen, X. Lan, J. Tang, J. Zhang, *Adv. Sci.* **2020,** 7, 2000195.

[4] D.Yuan, *ACS Appl. Mater. Interfaces.* **2020,** 12, 38333.

[5] L. Huang, H. Ye, W. Xiang, H. Fan, X. Liang, *J. Mater. Chem. C.* **2023,** 11, 8524.
